# Is a Forensic Cohabitation Program Recovery-Oriented? A Logic Model Analysis

**DOI:** 10.3390/ijerph19010009

**Published:** 2021-12-21

**Authors:** Shu-Ping Chen, Wen-Pin Chang, Bryan Fleet, Santoch Rai, Steve Panteluk, Alberto Choy, DeAnn Hunter

**Affiliations:** 1 Department of Occupational Therapy, Faculty of Rehabilitation Medicine, College of Health Sciences, University of Alberta, Edmonton, AB T6G 2G4, Canada; 2 Stepping Stones Behavioral Solutions, LLC, Indianapolis, IN 46205, USA; chang.wenpin@gmail.com; 3 Alberta Hospital Edmonton, Edmonton, AB T5Y 6A8, Canada; Bryan.Fleet@albertahealthservices.ca (B.F.); Santoch.Rai@albertahealthservices.ca (S.R.); Steven.Panteluk@albertahealthservices.ca (S.P.); Alberto.Choy@albertahealthservices.ca (A.C.); deannhunter@shaw.ca (D.H.)

**Keywords:** inpatient, tension, program evaluation, value-based practice, risk management

## Abstract

Background. Recovery orientation is a movement in mental health practice. Although general mental health services have taken the lead in promoting recovery, forensic psychiatric systems have lagged behind because of the need to reconcile recovery principles with the complexities of legal mandates. Advocating recovery and making systemic changes can be challenging because they require seeking a balance between the competing duties to the patient and the public. This paper used a logic model framework to demonstrate a cohabitation program that placed a woman and her newborn infant in a secure forensic rehabilitation unit, and analyzed the key assumptions of recovery upon which it was based. Methods. This was a qualitative program evaluation. Data collection involved individual interviews with the woman, the infant’s father, five primary healthcare providers, and five system administrators, and 11 focus groups with unit staff and other patients. Content analysis was used to guide the data analysis and develop the critical components of the program logic model. Results. A logic model that consists of input (team building, program planning, staff and patient preparation, resource management), output (logistic activities, risk management, mental healthcare, staff/other patient support, discharge preparation), and outcome (individual, provider, system, and society) components was developed. Conclusions. This study demonstrates a recovery-oriented program for a woman cohabitating with her baby in a secure forensic psychiatric rehabilitation unit. The logic model provided a comprehensive understanding of the way the recovery principles, such as shared decision-making, positive risk-taking, informed choices, and relational security, were implemented.

## 1. Introduction

Recovery-oriented practice has become a focus in mental health systems. “Recovery” refers to the ways in which a person with a mental illness experiences and manages a disorder in the process of reclaiming their meaningful life in the community [1]. Contemporary perspectives on the recovery-oriented practice highlight the support and development of personal control and responsibility, self-management, the capacity to change, quality of life, meaningful engagement, and social niche [2]. However, what is considered to be recovery-oriented and client-centered practice can appear to be in conflict with the context and structure of the forensic system. In particular, the service provided within forensic settings requires creative problem-solving between the patient and the treatment team in order to promote maximal participation in meaningful life roles and, at the same time, to abide by legal conditions. Institutional environments, out of necessity, exert considerable control over the daily activities of patients and constrain opportunities for social engagement. Therefore, the complexities and tensions within the forensic context should be addressed carefully when incorporating recovery concepts into inpatient services.

In this paper, we used a logic model framework to demonstrate the way that a cohabitation program for a woman and her newborn, in a secure forensic psychiatric rehabilitation unit, can be developed, and we evaluated whether the process and outcome promoted recovery in order to inform future practice.

Building a logic model is a method for designing, planning, evaluating, and implementing programs and it has been applied across diverse settings and disciplines [3,4,5,6]. A logic model is a theoretical framework that depicts the relationships between the processes in a program’s investment, delivery, activities, associated outputs, and outcomes [7,8]. A basic logic model consists of three core components: inputs, outputs, and outcomes. The inputs are the resources, such as human and financial resources, and the other efforts necessary to support program activities and produce outputs. The outputs are the processes, activities, events, and actions needed to implement the program, while the outcomes are the benefits or positive changes generated through the program, which can be measured as short-, medium-, and long-term outcomes [9]. Using a logic model in program evaluation helps ensure that evaluative thinking is integrated into the evaluation’s design and implementation [10]. We used the structure of a logic model systematically in order to evaluate if the program achieved the goal of promoting recovery. The findings of the evaluation can help our stakeholders reflect upon whether the cohabitation program resulted in the recovery-oriented outcomes desired [4,11].

We begin our paper with a brief overview of the program, followed by the methods, which outline the development of the logic model framework. Next, we present the program logic model, including what we invested, performed, and achieved. Finally, we discuss recovery orientation using the logic model to identify the assumptions, activities, and outcomes indicative of recovery-oriented practice, and the factors that facilitated and impeded the process and goal achievement.

### The Context and Setting of the Cohabitation Program

The cohabitation program was developed within the Northern Alberta Forensic Program, Alberta Hospital Edmonton, Canada, which operates at the interface of psychiatry and the law. It provides assessment and treatment services for individuals with unmet mental health needs who come in contact with the law, and the services include assessments for courts and rehabilitation under the auspices of the Alberta Review Board. A large part of the forensic program consists of rehabilitating individuals who have been found “not criminally responsible” (NCR) by reason of mental disorder. An NCR verdict is neither a conviction nor an acquittal, but, rather, it is a third alternative for the courts [12]. The large majority of NCR individuals have a severe mental illness in the form of psychotic spectrum disorders [13]. They cannot be sentenced in the usual way because they are not convicted of a crime and, therefore, the courts order them to reside in custody in a secure forensic hospital, although some may be allowed to remain living in the community from the outset, with legal conditions.

The legal expectation is that NCR patients are to be gradually reintegrated back into society, which involves transitioning to a conditional discharge, and, eventually, an “absolute discharge” from all legal obligations. This legal journey is overseen by the provincial Review Boards, which are quasi-judicial bodies that are required to review NCR patients, usually annually, in order to consider granting an absolute discharge if they are no longer a “significant threat the safety of the public” [14] (section 672.54). Therefore, the ultimate aim of the NCR scheme is not punishment, but providing “…sensitive care, rehabilitation and meaningful attempts to foster their participation in the community…” [12]. However, most community and hospital facilities are considerably challenged when pressed to accommodate the needs of a pregnant NCR woman with a stated goal of caring for her child. Therefore, this cohabitation program was designed to fulfill such needs. The NCR patient for whom the cohabitation program was developed was, at the time, a 28-year-old single woman, who discovered her pregnancy in late 2017, and delivered her baby in May 2018. She had an eight-year history of schizoaffective disorder and a history of medication noncompliance, substance use disorders, and interpersonal violence. She had an average intellect and essentially normal psychosocial functioning when mentally well, although she had persistent borderline personality traits. The charges that led to her NCR finding were assault with a weapon in 2016, although she also had a history of arson but no charges. Psychosis, precipitated by medication noncompliance and/or substance use, was associated with her history of violence. The Court determined her NCR status in 2016 by reason of her schizoaffective disorder. After her admission to the forensic hospital, she was compliant with psychiatric medications, but used street drugs intermittently, although this did not lead her to relapse into psychosis. With respect to her schizoaffective disorder, she remained in remission for approximately two years, and in remission for substance use for approximately one year at about the time of the cohabitation program (October 2017 notification of pregnancy and start of planning, with a May 2018 birth). At the time, she had one daughter, aged approximately 7 years, who was in her father’s custody. In May 2018, she gave birth to her second child—a healthy baby boy—while she was a forensic inpatient in a secure hospital. She had a prior diagnosis of polycystic ovary syndrome (PCOS) and she declined to use contraception, as she had incorrectly believed her PCOS had rendered her infertile.

Because of her legal status under the Review Board, she was deemed unable to live independently in the community immediately after she gave birth. Although this decision was primarily necessary because of her NCR status, specific risk factors that informed this decision included her history of violence, schizoaffective disorder, substance use, noncompliance with medication, as well the elevated risk of postpartum psychosis in the days and weeks immediately after birth. However, in light of her prolonged mental stability and good compliance in the preceding 1–2 years, as well the absence of functional disabilities, she was deemed able to parent her newborn baby in a staff-supervised setting.

Therefore, the forensic psychiatry service developed a cohabitation program that would allow the woman and her baby to cohabitate safely on a forensic inpatient unit. This program, consistent with the provincial health service’s policy, already in place, to allow a “Well-Infant” to cohabitate with an inpatient mother, was the first cohabitation program on a forensic psychiatry inpatient unit in North America, as far as we are aware, although a similar model was in place at the Edmonton Institution for Women. With respect to the medical duty of care, the mother was the “patient” on the unit and the well-infant was her sole responsibility and was considered a “visitor” on the unit. The assessment of the infant’s safety, and the ultimate decision to allow the infant to reside on the inpatient unit in his mother’s custody was, and remained, that of Child and Family Services, in collaboration with the hospital administration and the clinical team. The program lasted for about 4 months, starting when the baby was born, until the mother and baby were transitioned to the community mental health service.

## 2. Methods

We used a qualitative research approach to develop the program logic model, which guided the program evaluation by identifying key program components and illustrating the ways in which these critical components are related. The data collection activities involved 12 individually semistructured interviews, and 11 focus group interviews with all of the stakeholders, including the woman, her primary healthcare providers, the unit/hospital administrators at different levels, the infant’s father, and other patients in the unit, during October–December 2018. We obtained informed consent for participation directly from the mother and the infant’s father. Other participants were recruited by distributing posters and the study information in the team meetings (for healthcare providers), in the unit town hall meetings (for inpatients), and in the system administration meetings (for administrators). Those who were interested in participation would contact the researchers directly. The purpose of the interviews is to understand the social climate, the therapeutic regimes, and the program impacts. We seek to build on and promote further successful cohabitation programs by not only considering the patient’s perspective, but, more explicitly, also by exploring the experiences of and impacts on providers, system administrators, and other patients in the unit. The intention is to answer the following questions: (1) What is the understanding and impact of the cohabitation program from the perspectives of all stakeholders at the system, staff, patient, and environmental levels? (2) How do they see the benefits and challenges in cohabitating the mother and newborn in the unit?

Each interview lasted approximately 60 min, and each focus group interview lasted approximately 60–75 min. Their purposes were to understand the cohabitation program’s effects on all of the unit stakeholders. All interviews were audio-recorded and transcribed verbatim. The Research Ethics Board of the university approved the study procedure (Study ID: Pro00084270). Written informed consent was obtained after the procedure had been fully explained.

The following procedures were used to analyze the data from the individual and focus group interviews to develop the program logic model. First, we used open and incident-by-incident coding to label opinions, events, conditions, or phenomena, on the basis of the recovery concepts. Second, we used categorizing and axial coding to group conceptually similar codes to form categories and subcategories using more abstract headings. Third, relevant categories were organized to develop the structural and process components with a description of the program’s operational practices, with a specific focus on recovery-oriented practice. These critical components were integrated into a logic model diagram. Lastly, a member-checking exercise was used to establish the model’s credibility and face validity. This process occurred when the analytic categories and interpretations were tested with certain members of the groups who generated the data originally. A preliminary description of the program model and its critical domains were discussed in a meeting in which the participants were invited to share their opinions on the model’s structure and content. The final critical components were amended on the basis of this exercise.

## 3. Results

**Figure 1:**
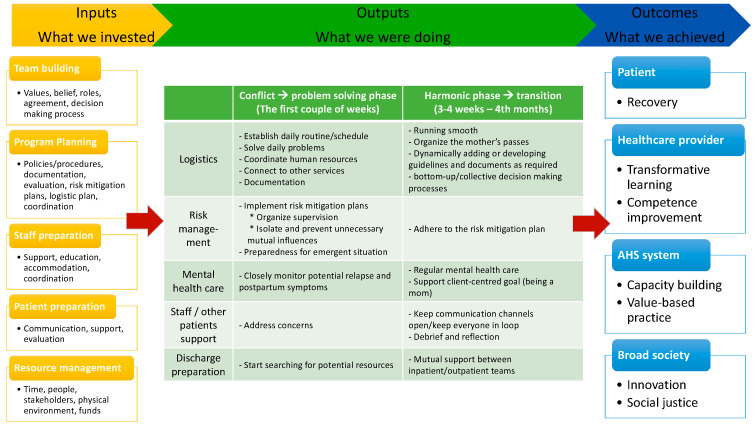
The program logic model.

Five system administrators, the woman, the infant’s father, and five primary care providers—three nurses, a psychiatrist, and an occupational therapist—were interviewed individually. Three focus groups, which consisted of 12 inpatients in the unit, and eight groups, which consisted of 21 multidisciplinary frontline providers, were conducted as well. Figure 1 depicts the logic model developed on the basis of the thematic analysis of the interview data.

### 3.1. Input Aspects

The input aspects, which represented what we invested before the program, consisted of five components: (1) Team building; (2) Program planning; (3) Staff preparation; (4) Patient preparation, and (5) Resource management. Team building was the first step to reaching a collective agreement, identifying the key values and beliefs of the cohabitation program, and establishing a decision-making process. During the team-building process, recovery was identified as the core value and philosophy that guided the program’s direction. As an administrator highlighted: “Our ethics of being least onerous, but also being recovery oriented, we felt that we needed to explore this option [recovery]” (ADMIN 3). A provider also echoed: “We’re promoting recovery and this is a unique situation where recovery for this individual is very important in a special way and that requires us as a treatment team to actually facility that” (PHP 4). Effective communication was key at this stage to reach common agreement, as ADMIN 5 shared: “It was more tweaking and communication and making sure that what we were doing was still recovery-based and within the values of [the system], … So, it was a lot of stakeholder engagement and communication.” The identification of recovery as a core value was a key determinant in the program’s advancement.

In the program planning, it was necessary to first review, adjust, or develop new policies, procedures, or guidelines in order to implement the recovery-oriented cohabitation program. Further, the program planning involved a feasibility evaluation, risk assessments, and a plan for any logistics that adhered to the recovery principles. Provider 5 commented: “In fact, when the plan was actually put to management, all of the comments I got was that, ‘Well it seems very recovery-oriented.’ So that was the language that was used and so that was what was driving [the program].” In the program preparation stage, it was crucial to prepare the staff by providing support and education to enhance their experience and understanding of recovery in a forensic context. A provider noted:

We’re moving more towards a patient-centered approach to forensic care, a more recovery orientated approach towards forensic care which is driven by the goals of the patient, and the fact that the clinical staff on the unit are moving more towards that and there’s been, in recent years, a lot of education about recovery and what it means in forensic; and so that’s kind of laid the groundwork for people to start thinking outside of the box.

(PHP 4)

In addition to education about recovery, the staff also received support and training to equip them with the skills to manage new issues, such as pediatric-related critical responses, which are usually outside the practice of mental health professionals.

Other patients on the forensic unit were undergoing their own unique recovery journeys, and it was important to ensure the quality and safety of their individual rehabilitation and reintegration into the community. Therefore, it was essential to have an open conversation and ongoing communication with other patients on the unit and provide support to address their potential anxiety and concerns, as well as to minimize any tensions or misunderstandings that may arise between people on the unit. An administrator shared:

I think for the patients it was empowering to know that when you trust your treatment team and you work with them and say, this is something that’s important to my recovery, they hear you. They don’t just say no because logistically it’s challenging and no because it hasn’t been done before. They hear you, they work with you and they help you achieve your goals.

(ADMIN 3)

With respect to resource management and attention to results, it was essential to ensure that frontline staff, all physicians, community partners, and other stakeholders were committed to the program and that they worked as a cohesive team.

### 3.2. Output Aspects

The output aspects, which represented what we performed during the program, consisted of five activities, conducted in two phases: (1) Logistic activities; (2) Risk management; (3) Mental health care; (4) Staff/other patient support; and (5) Discharge preparation. When the baby arrived on the unit, the first 2–3 weeks of the program were identified as the first phase, during which daily conflicts occurred and problems were solved. The second phase began when daily routines were established, harmony was achieved, and the transition plan was initiated.

Logistic activities included, but were not limited to, establishing a daily routine/schedule, coordinating services in the first phase, and developing guidelines and documents as required in the second phase. Positive risk-taking is one of the recovery principles. As ADMIN 3 explained: “We’ve always had, in my opinion, a group that really had a strong understanding of recovery-oriented principles and really had a strong understanding of positive risk taking and also a willingness to try.” Therefore, risk management and risk mitigation plans were a key consideration in the program. These needed to be in place and implemented when possible, and the staff needed to be trained as well to prepare for emergent situations. As Provider 4 noted:

I think yes, we did take risks, that we did need to take risks to promote recovery, and at the same time I think what really made it work was there was a lot of trust between all the parties; so, I think that really helped.

The other significant activity during the program was providing mental health care to the woman. In addition to delivering client-centered and recovery-oriented care throughout the program, it was particularly important to monitor potential relapse and postpartum symptoms during the first phase.

Addressing concerns, maintaining open communication, and debriefing and reflection were three critical aspects of providing support to staff and other patients in the unit. Such support played a key role in reducing the tension on the unit and improved the ward climate. For example, a patient shared:

I trusted in the actual assessment of the treatment team… they know each patient individually quite well. So, I trusted that they had appropriate amount of patients and the right patients on the unit. And I felt that when the baby was actually around that everybody was on their best behaviour, almost, yes. There was, there was just like a calmness and peace to the unit that I haven’t felt before, and I’ve been in the system for five years.

(P2-2)

When the woman and her baby were ready for discharge, searching for potential resources and providing mutual support between the inpatient and outpatient teams were two main areas of discharge preparation.

### 3.3. Outcome Aspects

The outcome aspects, which describe what we achieved, varied according to the stakeholders. For the woman herself, the cohabitation program helped her advance her recovery through her strong bond with her newborn and improved her mental health and sense of wellbeing. An administrator explained further:

She’s [the mother] actually further along in her recovery journey, because she was able to achieve one of her major goals, which was being a mom. She says, “I want to be a mom,” then, you know what, we have to help you be a mom. So, I think that’s what we did. And, because we did that, she was able to continue her recovery in such a positive way.

(ADMIN 5)

The woman herself reflected on the program, and shared:

My recovery, my children have always been a protective factor for me. I fell into substance abuse when I didn’t have my daughter. So, being able to have a second chance and not make the same mistakes, he’s a huge protective factor, so, it’s been really good to have him [the baby], for sure, for my recovery.

Other patients in the unit also benefited from the program through their positive emotional, social, and behavioral responses to the patient, and gained a sense of being trusted as well. As one patient said: “It’ll be a lovely experience for everybody involved, right, so I figured that if, well the staff trusted us enough to have a baby on the unit, and that was pretty cool.” (P3-1). He emphasized:

I just want to say that was a very good experience for me. Like taking care of the kid, and watching her take care of the kid, you kind of like learn a little bit of how to take care of kids and stuff, so it was a learning experience for me.

(P3-1)

Further, the patients trusted the psychiatric unit team to act in their best interests; as a patient expressed:

I guess maybe just the whole idea of how much the rehabilitation program here at the Hospital… how they actually do care for our wellbeing. It really opened up my eyes to the whole idea that, you know, they really do have our best interests at heart.

(P2-2)

At the system level, the cohabitation program’s effects included the growth of the system’s capacity, flexibility, and risk tolerance, the creation of knowledge and a self-developed toolkit, and the patient and family-focused value-based practice. As ADMIN 1 shared, the lessons he learned were “…understanding how we can engage in client-centered recovery focused care in expanding the definitions and the roles and what we were able to do as a forensic service for our clients.” ADMIN 5 illustrated further the recovery-based values embedded in the program:

I’m just so proud of my team for going above and beyond for the patient. They were—had their head so in the right place and their hearts, and this was purely about recovery and best practice for patient care.

The frontline providers endorsed the sense of system flexibility and capacity building; as a provider expressed:

I think it’s kind of created perhaps a self-awareness that we can do things differently … it’s demonstrated that the system is open to change and that it’s open to new ideas and innovation and particularly along the lines or recovery, promoting recovery. I don’t believe it’s been a shock to the system. I think that it’s been an awakening or an understanding what the system actually is like, you know that it’s much more flexible and much more positive and much more patient-centered.

(P4-1)

Overall, the cohabitation program’s broader effects were that it showed the implementation of a client-centered, recovery-focused, and innovative program in forensic psychiatric services, as well as advocated for social justice and equity.

The issues and challenges in delivering recovery-oriented services in a forensic inpatient rehabilitation setting were also raised. As ADMIN 1 described the unit: “The context of that within forensics is actually very difficult, because client-centered and more specifically recovery oriented, some of the natural principles are in direct contradistinction to the actual structure of our program, you know, which is custodial and detainment.” This leads to problems in developing a shared meaning of recovery within the context. As we understand that fundamental components of recovery practice are person-centered and include empowerment, transferring these components of practice into an inpatient setting has certain difficulties. Tensions occurred between maintaining order on the unit and ensuring the choices, preferences, rights, and satisfaction of patients. As a provider shared her observation of the mother:

I guess I just want to kind of recognize my own tension with being watched all the time. … it’s tough to deal with, you know, like a person you wouldn’t ever imagine—a regular human being observed all the time… You just want to, some days you just want to have your own space, your own time to yourself, and I do get it...”

(P2-3)

All providers expressed the importance of the patients’ choices and decision making, but conceded that, in the real world, they have to manage potential risks to ensure patient safety and prevent any harmful consequences. During the program, it was found that frontline providers underwent a learning process that transformed their practice from risk-averse and custodial strategies into one that supported opportunities to engage and take responsibility. Another lesson the providers learned was to be more comfortable sharing power with patients. The majority of providers agreed that they were well-rounded in client-centered and recovery-oriented services by building “real, trustful relationships with patients” (P5-5), advocating for their patients, and resolving the conflicts raised in recovery-oriented daily practice.

## 4. Discussion

This paper describes a logic model for a cohabitation program that forensic psychiatric inpatient services throughout Canada can use. The qualitative data collected in this specific forensic psychiatric unit were instrumental in creating the logic model’s components and for achieving insight into the particular inputs, activities, outputs, and outcomes needed to reach the overarching goal.

When considering both recovery and forensic settings, it is important to reveal both the individuals’ and the services’ perspectives on risk. Forensic service users have committed a crime that usually is considered sufficiently serious to require their confinement in a forensic service facility. They are often assumed to pose risks of harm to others, compromise security, and demonstrate a considerable level of aggression and violence. This stereotype has a profound effect on the design of the forensic mental health environment, which emphasizes security and risk prevention. In this respect, recovery constitutes challenges in forensic services [15]. The way the emphasis on risk in secure services exerts an influence on recovery-oriented practice has been discussed in a growing body of recovery literature [16]. The forensic services need to acknowledge risk-taking as a fundamental part of personal growth and learning, and the perception of the individual’s risk of offending must be balanced against their opportunities to recover [17]. The other example is the relational security model [18], within which security is viewed as a process of risk management with three components: the physical aspect of security and control, policies and procedures, and staff–service-user relationships. Our logic model, including patient communication and support in the input and output activities, highlights the quality of the therapeutic relationship and the way that mutual trust between staff and service users affects security and changes in risk behaviors. Through recovery-oriented policies and procedures developed in the program, the logic model also demonstrates recovery as the foundation for change and for promoting safety.

From the personal perspective, moving beyond a criminal self-identity is key to the recovery of forensic service users [19,20]. In our case, the woman’s redefinition of herself, and her rediscovery of a positive sense of self as a mother, rather than as an offender and mentally ill person, was a significant task in her recovery journey. As she highlighted in the interview, the program provided her an opportunity to build a life role beyond illness and criminality, and she perceived that the cohabitation program, which allowed those self-changing processes, was recovery-promoted. Motherhood engendered her hope, increased her self-esteem, and maintained her social functioning in her life. We learned from the program that the recovery of forensic service users has additional complications involving personal guilt and social influences. Recovery-oriented practices in forensic settings need to address these complicating factors and focus on interventions that help people find a new identity and meaning in life.

Services users in forensic settings often perceive treatment as more coercive than in other mental health settings [21]. Because of the association between offending behaviors and mental illnesses, it is always difficult to achieve a balance in forensic settings between offering choices and the potential for harm, while promoting hope for progress toward a meaningful life. By providing service users choice, our cohabitation program created a safe environment in which all patients felt that their steps toward recovery were possible and supported by the treatment team.

Recovery is promoted when frontline providers are equipped with the ability to help patients become actively involved in their own lives. In this study, our providers built upon, and were motivated by, the belief that the woman was able to find a path to her recovery. They supported her aspirations, hopes, and needs to play the role of a mother. However, in the “investment” (input) stage of the program, the question about the workforce’s understanding of recovery principles was raised, and a series of education and empowerment activities was planned, in which staff had ample opportunity for discussion and questions. Particularly, the questions of risk and choice were debated hotly with concerns about “what if” scenarios. Nevertheless, these set the stage for staff acceptance and also highlighted the challenges in changing the practice culture. Thereafter, staff were able to identify recovery as the core of valued-based practice and to think of a “real” client-centered approach. They learned to be open, but clear, about limits. They appreciated the collaborative decision-making process and remained sensitive to the individual’s needs. Some staff in this study described the program as a personally meaningful and transformative learning process, in which changes in their attitude influenced their practice. We believe that building such awareness was fundamental to the program’s success.

However, recovery-oriented practice at the provider-level cannot occur in the absence of broader organizational support and facilitation. In this study, we found that, although the clinical team became more educated and focused on recovery, the program could not have been implemented successfully had the same ethos not been embraced at the system level. Essentially, what occurred was that the system administration assigned an equal value to patient recovery. A single program cannot demonstrate full support for recovery. It involves reflecting on the way the entire system views mental health problems and the role of forensic psychiatric rehabilitation. To practice recovery-oriented services in forensic settings “safely” and effectively, it is necessary to reconceptualize service approaches fundamentally, at the system level. Hence, the organization’s role, new approaches to intervention planning and programming, and the evaluation of patient outcomes had to be redefined. Our program demonstrates leadership’s imperative role in promoting recovery by providing sufficient participation in decision making and access to resources.

Organizational support, particularly for risk-management planning and new practice guidelines, must be clear and transparent and involve collaboration and discussion with frontline staff. A clear structure and support for staff on the part of all administrative levels is necessary to facilitate the adoption of the new recovery-oriented approaches.

The overarching goal of developing this program logic model was to provide all of the inputs and implement the activities in order to achieve a series of outputs and outcomes for the cohabitation program. Notably, through this model, outpatient stakeholders, such as Forensic Assessment and Community Services (FACS), will be able to provide similar services in the community to support this woman’s wellbeing and recovery upon her transition from inpatient services. The model developed here can be used as a reference for future program development and implementation, as a tool to engage stakeholders, and as a framework to guide program evaluation. We illustrated how forensic psychiatric services can share recovery values and practices in inpatient contexts. To promote recovery movement, the future direction for forensic psychiatric services should address the ethical tensions between the fundamental principles of recovery and their moral considerations in practice.

One limitation of this model is that the components were measured only qualitatively, while a more rigorous mixed-methods approach may have provided more detailed findings. In addition, it is critical to recognize that the components required may not be the same for all NCR patients. Future research should examine the differences in the logic model components’ results if a different research method is used. In summary, this model may serve as a framework to provide cohabitation services for both NCR pregnant patients and their infants.

## 5. Conclusions

The process of providing recovery-oriented services in secure forensic settings is complex. This study demonstrates the ways that services could implement a recovery orientation through a cohabitation program model that reflects both recovery values and a forensic awareness of safety and security. The program provided the woman opportunities to build a life beyond illness and to find motherhood a meaningful occupation in her recovery journey. Although the recovery journey of people in forensic services differs somewhat from that of others, application of the recovery principles, such as patients’ participation in their care, shared and transparent decision making, positive risk taking, informed choices, and relational security among all stakeholders, were evident in the program logic model.
